# Self‐determination of people with profound intellectual and multiple disabilities

**DOI:** 10.1111/dmcn.15363

**Published:** 2022-07-22

**Authors:** Synne Nese Skarsaune

**Affiliations:** ^1^ VID Specialized University Centre for Diaconia and Professional Practice Norway

## Abstract

Self‐determination is a human right that people with profound intellectual and multiple disabilities (PIMD) risk not being granted. Exploration of such topics and research in general has traditionally not included people with PIMD as sources of knowledge; rather, the perspective of others has been sought. Ethnographic methods highlighting descriptions of lived experience have been argued as a way of including such individuals, producing knowledge building on the person's perspective. Exploring the human right to be self‐determined through ethnographic approaches can bring novel ways of understanding the concept, both about how to listen and learn from such experiences, and about implications for understanding self‐determination. All people have the potential for self‐determination, by being understood through embodied communication in caring relationships. By fostering relations with people with PIMD in sensitive, ethical ways, and addressing their profound dependency, their self‐determination can be realized.

**What this paper adds:**

- Attentive engagement with people with lived experience of profound intellectual and multiple disabilities can inform both researchers and clinicians on self‐determination.
- Through real‐life descriptions, self‐determination is demonstrated to move beyond independence and choice‐making.

## What this paper adds

- Attentive engagement with people with lived experience of profound intellectual and multiple disabilities can inform both researchers and clinicians on self‐determination.
- Through real‐life descriptions, self‐determination is demonstrated to move beyond independence and choice‐making.

This article's abstract has been translated into Spanish and Portuguese.

Follow the links from the abstract to view the translations.

Plain language summary: https://onlinelibrary.wiley.com/doi/10.1111/dmcn.15558

## ResumenHabilidades motoras 'normales‐bajas' en bebés con alto riesgo de trastornos del desarrollo: un estudio de prevalencia y pronóstico

**Objetivo:**

Investigar la prevalencia y el valor pronóstico de las habilidades motoras "normales bajas" en bebés con alto riesgo de trastornos del desarrollo.

**Método:**

Los bebés nacidos con un peso extremadamente bajo al nacer y extremadamente prematuros dados de alta de la unidad de cuidados intensivos neonatales entre 2015 y 2018 completaron la Escala motora infantil de Alberta (AIMS), Evaluación del desarrollo motor neurosensorial (NSMDA) a la edad corregida de 4, 8 y 12 meses. y la Escala de Desarrollo Mental de Griffiths a la edad corregida de 12 meses.

**Resultados:**

Los lactantes participantes (n = 191) con una edad gestacional media (intervalo de confianza [IC] del 95 %) de 26,80 semanas (26,60, 27,1) y un peso al nacer medio (IC del 95 %) de 869 gramos (843, 895) incluyeron 45 (23,80 %) lactantes pequeños para la edad gestacional. La AIMS calificó al 50,32 %, 35,37 % y 14,86 % de los bebés dentro del rango de habilidades motoras "normal‐baja" (1‐2 DE por debajo de la media para la edad) a los 4, 8 y 12 meses, respectivamente. De los bebés dentro del rango de habilidades 'bajo‐normal' de AIMS, el 55,70 %, el 88,46 % y el 59,10 % fueron clasificados como afectados por la NSMDA a los 4, 8 y 12 meses, respectivamente. La evaluación de Griffiths a los 12 meses identificó solo el 7,33 % de los bebés con habilidades "normales bajas" y el 3,33 % con discapacidad motora. La calificación de deterioro motor mínimo en la NSMDA a los 4 u 8 meses predijo significativamente el desarrollo general a los 12 meses.

**Interpretación:**

Los bebés de alto riesgo con habilidades motoras "normales‐bajas" pueden justificar la derivación a una intervención temprana, ya que el deterioro asociado representa un mayor riesgo de trastornos del desarrollo general más deficientes.

## Resumo

**Objetivo:**

Investigar a prevalência e o valor prognóstico de habilidades motoras "abaixo‐do‐normal" em bebês de alto risco para pobre desenvolvimento.

**Método:**

Bebês nascidos de extremo baixo peso ao nascer e extremamente prematuros que receberam alta da terapia intensiva neonatal entre 2015 e 2018 e completaram a Alberta Infant Motor Scale (AIMS), Neuro‐Sensory Motor Developmental Assessment (NSMDA) na idade corrigida de 4, 8 e 12 meses, e Griffiths Mental Development Scale aos 12 meses de idade corrigida.

**Resultados:**

Participaram 191 bebês com idade gestacional média (intervalo de confiança de 95% [IC]) de 26,80 semanas (26,60; 27,1) e peso médio ao nascer (IC 95%) de 869 gramas (843, 895), dentre estes 45 (23,80%) lactentes eram pequenos para a idade gestacional. A AIMS pontuou 50,32%, 35,37% e 14,86% dos bebês dentro da faixa de habilidades motoras "abaixo‐do‐normal" (1‐2 DP abaixo da média para a idade) aos 4, 8 e 12 meses, respectivamente. Dos bebês dentro da faixa de habilidades "abaixo‐do‐normal" da AIMS, 55,70%, 88,46% e 59,10% foram classificados como tendo prejuízo pelo NSMDA aos 4, 8 e 12 meses, respectivamente. A avaliação de Griffiths aos 12 meses identificou apenas 7,33% dos bebês com habilidades "abaixo‐do‐normal" e 3,33% com deficiência motora. A classificação de disfunção motora mínima no NSMDA em 4 ou 8 meses previu significativamente o desenvolvimento geral em 12 meses.

**Interpretação:**

Bebês de alto risco com habilidades motoras "abaixo‐do‐normal" podem justificar o encaminhamento para intervenção precoce, pois o comprometimento associado representa um risco aumentado para desfechos pobres no desenvolvimento geral.

**Abbreviation**
PIMD: profound intellectual and multiple disabilities.

People with profound intellectual and multiple disabilities (PIMD) have traditionally been excluded from research, being regarded as too challenging to include.^1^ There is thus a scarce knowledge base that includes people with PIMD.^2^ Several methodological challenges have affected progress in the field,^3^ including issues related to informed consent.^4^ Research has been based primarily on the views of significant others, for example staff or family.^5^ Such studies provide valuable contributions, but the field still lacks the perspective of the very individuals it studies. There are exceptions, though, and a body of articles that are grounded more explicitly in the lives of people with PIMD will be presented in this review. These articles aim to make the person ‘more than just the object of the researcher's gaze’.^2^ These people are not able to verbalize views in formal ways, necessitating a more indirect way to portray their perspective. This might be done by description of their lived life through a researcher or a significant other's close details. Although recognizing that this involves elements of interpretation through the person writing or giving the description, and where the individual with PIMD cannot validate with verbal words, such efforts might be of value. It has been argued that ethnographic methods are suitable for this.^6^ Ethnographies can be conducted in many ways, including the use of, for example, observations and interviews. Common for different approaches is the aim of giving voice to people in their own local context, often relying on ‘thick’ descriptions of events.^7^ It is important to keep an open mind about the people under study. For those with PIMD, ethnographic approaches, particularly through close observations, might thus contribute to knowledge that might otherwise be left unknown.

Self‐determination is a human right highlighted in the Convention on the Rights of Persons with Disabilities,^8^ which calls for the respect of inherent dignity and individual autonomy of all people. Practice, however, shows that people with disabilities – especially those who are profoundly dependent – risk not having this right respected.^9^ People with PIMD are a heterogenous group, but they share some common traits, including little or no apparent understanding of verbal language, almost no ability for self‐support, and an array of sensory impairments.^10^ The lives of people with PIMD are marked by profound dependency.

Within Western philosophy, rationality has often been linked to moral worth, thus leading to the disqualification of claims of self‐determination of people with cognitive impairment.^11^ Even in a more ordinary understanding, people with PIMD have been devalued and assumed unable to experience self‐determination.^12^ This assumption is linked to self‐determination traditionally being equated with independent choice‐making.^13^ The concept's history within disability, emerging from the 1990s, has emphasized a causal agent making choices.^14^ The disability movement has striven to enhance independence and allow people to be agents in their own lives, counteracting paternalistic services.^15^ In line with this, studies have also examined the potential for independence among people with the most profound needs, for example through the use of different technological devices^16^ or making choices through preference assessments.^17^ However, it might be argued that viewing self‐determination as solely independent choice‐making risks overlooking the full potential of people with a profound need for support.^18^ This review suggests a more inclusive and broader view of self‐determination, addressing the topic through arguments grounded on the possible perspective of the people with PIMD.

The inclusion of people with PIMD in research involves methodological challenges that will be reflected upon. Two issues are particularly prominent: how the life of the person with PIMD is depicted through ethnographic descriptions, and how these contribute to further ethical and philosophical reflections upon the topic of self‐determination. These two concerns, moving from real‐life narrations towards a more ethical and philosophical domain, suggest ways of learning from people with PIMD about self‐determination. This research contributes to the knowledge base on self‐determination, which is significant for clinicians working closely with people with PIMD; moreover, while such research is difficult, it may inspire future research to address this perspective. This somewhat unexplored approach may broaden understandings and highlight conditions for listening to and learning from people with PIMD. The rationale of the included articles is that to be able to say anything on the topic of self‐determination for people with PIMD – or other topics, for that matter – the person concerned must be consulted.

## METHOD

Guiding points from Ferrari^19^ were applied when performing the review. Even though this was not a systematic review, a search was made in EBSCO and Scopus databases, scoping the target group of people with PIMD and the topic of self‐determination, delimited to peer‐reviewed articles from 2000 until January 2022. This broad search generated a total of 658 articles. Adding the criterion of ethnographic approaches, reviewing the abstracts for an explicit focus on self‐determination generated a very scarce body of literature (*n* = 3). Therefore, a broadened, implicit engagement with self‐determination was included, searching for articles that explored how to obtain the perspectives of people with PIMD, or applying the intertwined concepts of agency, personhood, or citizenship (Figure S1). These approaches are connected to understanding a person and that person's preferences – a necessary precondition for self‐determination. The three main inclusion criteria – relating to people with PIMD, having a broad focus on the topic of self‐determination, and using an ethnographic approach – yielded six articles representing some form of ethnographic material involving close observation.^20^, ^21^, ^22^, ^23^, ^24^, ^25^ Two more articles were included,^26^, ^27^ although they did not follow the strict design of an empirical study but rather offered philosophical reflections upon themes closely linked to self‐determination. These articles were included because their arguments were grounded in detailed descriptions of the lives of people with PIMD provided by their parents, much like ethnographic descriptions. As such, all eight included articles represent attempts to include people with PIMD through real‐life descriptions in ways that make them sources of knowledge (Table 1).^2^

**TABLE 1:** Details of included articles

Reference	Engagement with self‐determination	Number of cases	Consent	Method	Observational data	Demonstrations of real‐life descriptions pointing towards self‐determination
Boahen^20^	Implicitly, through exploring how to get hold of the person's perspective	1	After assessing ability to consent, his parents were appointed as consultees	Ethnographic case study involving observations	Observing and describing the relationship between Abrax and the researcher, and his relations with his supporters – through everyday living	‘Abrax used monosyllabic sounds to communicate and required assistance with all aspects of his care. There was a hermeneutic relationship between him and his carers before a judgement was made about what his utterances meant. So for instance if they asked him “Abrax do you want to eat?” he would make sounds that they interpreted and relayed to him, which he responded to until some agreement was reached. Thus Abrax and his carers displayed a relational model of decision‐making because they depended on each other to achieve shared meanings of the world’.
Mietola et al.^21^	Implicitly, through exploring how to get hold of the person's perspective	6	Proxy and continuous process of assent, constantly evaluating participants well‐being during fieldwork	Ethnography: observations and interviews with family members and staff	Observation in everyday living (at home, day centres, other contexts of everyday living)	‘Anna is driving a motomed (a motorized stationary bike) in the living room. After driving a while, Anna starts to cry. There are no tears, but she opens her mouth and moans quietly, and there is an unhappy expression on her face. It seems to me that she probably would like to stop driving motomed’.
Simmons and Watson^22^	Implicitly, through the concept of agency	1	Proxy and ongoing consent sought from Sam, monitoring whether he was happy to be in the researcher's presence	Pre‐observation focus groups, observations ongoing, dialogue with staff and parents	Participatory and non‐participatory observation. The use of vignettes	‘Sam is sat on his artificial grass mat on the carpet for registration. One of Sam's neighbours stands up, walks over to a yellow box in the corner near the teacher, pulls out his switch and returns to the carpet. She tells Sam to press his “blue button” and smiles. Sam leans forward. The girl takes Sam's hand and places it on top of the switch. The switch is activated and emits a pre‐recorded “Good morning!” message. Sam repeatedly hits the switch with both hands (he raises his hands, then suddenly slaps the switch held in front of him, lets his hands fall on his lap, and repeats several times). Sam presses the switch before the recorded message has ended, resulting in the first half of the message being played, over and over. Between each switch‐press Sam flaps his arms like a bird whilst smiling and vocalises (“Ooooooh!!!”)’.
Skarsaune et al.^23^	Explicitly, exploring self‐determination	2	Proxy and process consent, observing the people's reactions during observations	Ethnography: observations and interviews with family and staff	Close observations of the professional relationship between people with PIMD and professionals. Some use of video	‘After a couple of songs, carer Tiril find the micro‐switch and invites Vera to indicate that she wants more music by touching it with her chin. After a few somewhat reluctant efforts, Vera turns her head away from the switch. Sensitive to the situation, Tiril asks “Is this not what you wanted?” She moves the switch close to Vera's chin to give her another chance, but Vera remains motionless. You're quite right, Vera. You should be allowed to listen to music without having to press the switch’.
Stefánsdóttir et al.^24^	Explicitly, exploring relational autonomy	24	Proxy and close collaboration with staff and relatives to make sure observations were carried out with participant's consent	Observations and focus group interviews with staff	Observing everyday life in homes and daily activities	Gunnar signalled not wanting to go on the standing frame, by ‘making distressed sounds and facial expressions all through the exercise. Sometimes he cried and in an attempt to remedy the situation the staff played music for him and read to him, but without success’. At a meeting, other training forms were suggested and implemented. He showed his approval by smiling.
Watson et al.^25^	Explicitly, exploring supported decision‐making	5	Proxy and assent in the form of non‐verbal indications that the person of focus was comfortable participating in the study	Action research/iterative approach, implementing the framework of supported decision‐making. Triangulation of multiple data sources. Surveys, semi‐structured interviews, discussion groups, workshops, observations, and document review	Using an observation template and video‐taped data exploring responsiveness in relation between person with PIMD and supporters	Support group were to decide on whether Neil should have a tracheotomy due to aspiration pneumonia. Supporters reflected together, drawing on their relationships with Neil as well as his past life experiences, specifically his past experiences of tracheotomy. Jodee (house supervisor) recalled Neil's reaction to his most recent tracheostomy 3 years earlier: ‘I remember, you do too (looking at Neil's mother), he hated it. It was horrible. He was so distressed he seemed so scared. Remember.’ Neil's mother replied, ‘don't you worry love, I remember. I videoed him on my phone, it was such a God‐send when they got rid of it. If they hadn't Neil would have pulled it out himself, don't you worry about that’. The circle of support around Neil decided for him to not have the tracheotomy.
Kittay^26^	Implicitly, through the concept of personhood	1	Not addressed	Philosophical paper – building argument on the lived experiences with her daughter	Lived experiences	‘I have written quite a bit about her love of music, especially but not exclusively classical symphonic music, with the master of this form, Beethoven, being at the top of her list. So much for the assertion that persons with severe mental retardation cannot experience aesthetic pleasures’.
Vorhas^27^	Implicitly, through the concept of citizenship, dignity	3	Not addressed	Using close descriptions of lived life in philosophizing, provided by parents	Discussing the lives of three people in connection with a series of questions in moral and political philosophy	‘There are many things Inaaya can do: she can let me know that there are certain foods she likes, certain tastes she likes. If she likes something, she might bounce her arms up and down whilst she's sitting down, or rock and sway or kick her legs …’.

Abbreviation: PIMD, profound intellectual and multiple disabilities.

Articles were excluded if descriptions of the participants clearly indicated a higher level of functioning than that of people with PIMD, for example through the use of verbal speech. Articles were also excluded if data were primarily based on the perspective of others, such as interviews or surveys with staff or parents where close descriptions of lived experiences of the person with PIMD did not constitute the data material. Studies involving description from observations where the main aim was to measure a specific behaviour or the effects of interventions rather than a more open description of lived life were also excluded. Though interesting, books based on ethnographic or other material portraying the lives of people with PIMD^6^, ^15^ were excluded. Also, empirical studies using different creative methods that brought in the perspective of the person with PIMD through the use of photo‐voice^4^, ^28^ but focusing on themes other than self‐determination were excluded.

While not presuming to have covered the entire field of knowledge, this review may still contribute by shedding light on the field of self‐determination, demonstrated through relevant literature.

## THE PARADOX

It is an apparent paradox to do research on self‐determination that includes individuals who cannot formally consent. To implement research involves applying ethical guidelines, and the principle of informed consent has been positioned as the cornerstone of research ethics. The Helsinki Declaration^29^ argues that if a study includes people not capable of giving formal consent, then the benefits must outweigh the possible harms. To include these voices can be argued to counteract further marginalization and the silencing of people with PIMD:

it is actually unethical to exclude persons with PIMD from research that could provide insights about their subjective experiences … .^21^

To address the paradox, formal consent is normally provided by a proxy, although studies also apply sensitive ways of assessing a person's ongoing approval of research activities to evaluate possible harms. These might, for example, involve the researcher developing an understanding of the person‐specific way of being and communicating, and collaborating closely with people who know them well.^21^ With this background knowledge, researchers can pay attention to whether the participant seems comfortable with the researcher's presence, checking their understanding of the person's responses with caregivers, and preparing to withdraw if it is deemed that participants are disturbed.^23^ These suggestions demonstrate more holistic approaches to informed consent, focusing on the well‐being of the participants.^21^ The ethical demands on the researcher are great, and thorough reflection upon what situations to partake in is of upmost importance, balancing the value that a close description of a person's daily life might bring with respect for that person's privacy.^21^, ^23^, ^24^ The aim is to bring understanding about these lives, but in non‐intrusive and respectful ways.

## METHODOLOGICAL AND EPISTEMOLOGICAL IMPLICATIONS

To be able to present ‘thick’ descriptions, it is important to build trust and understanding of the person's distinct way of communication. This will often necessitate the researcher following a few people closely, rather than many. Typically, people with PIMD use pre‐symbolic, embodied communication, which requires a broadened view of meaning to grasp their intentions:

communication have [sic] to be viewed as being possible in all behaviour, not merely conventional forms of symbolic communication.^23^

The richness of embodied communication might, for example, be portrayed through the use of vignettes, offering ‘thick’ descriptive pieces. For example, the following excerpt describing a situation with Sam, a young person with PIMD, demonstrates this:

Sam is sitting on the carpet, leaning on several children, they are talking to each other and Sam appears to be listening, his mouth is open, his eyes are rolled back to the top left and his ‘good ear’ is facing in the direction of the conversation. He appears to be concentrating.^22^

However, even though we aim to stay close to what can be observed, there is often a need to supplement with interpretations, to suggest what bodies are communicating. Observational data might thus be supplemented with interviews with family or staff who know the person well, recognizing the researcher's limited possibilities of grasping the person's meaning.^20^, ^21^, ^23^, ^24^, ^25^ In this lies an epistemological assumption of knowledge as being co‐constructed, where the perspective of the person with PIMD must always rely on mediation. Mediation in the Vygotskian^30^ sense emphasizes the support needed to establish relations between the person and the world, a condition of being for a person with PIMD.^23^

Even though the literature is based on an optimistic view of including people with PIMD, the uncertainty embedded in such efforts is still recognized:

Despite the experience we gained during the fieldwork of our participants' means of communication, our interpretations remained partial and situational … .^21^

The literature calls for sensitivity about validity questions, being cautious not to favour the researcher's or one of the significant other's interpretations, but rather to explore understanding through plural voices. Further, communication might have different significance in different settings, thus necessitating rich contextualization.^22^

## SELF‐DETERMINATION UNFOLDED THROUGH ETHNOGRAPHIC DESCRIPTIONS

The literature provides a rich material of the life of people with PIMD, through which different suggestions of self‐determination might be given. For instance, there is a description of a young female's close interaction with a professional carer and what was understood as being met on a need to experience proximity in a stressful situation,^23^ as well as a rich description of a male being met and understood about his rejection of a certain form of training.^24^ Another example might be this description provided by a parent:

Inaaya has her own way of communicating. When she's happy she blows raspberries, and she communicates a lot with her feet and hands: when she's excited she will kick her feet, and when content she leans against me.^27^

The examples portray how non‐verbal embodied communication is interpreted as demonstrating the person's preferences and self‐determined will. These can also be communicated through what one does not like:

He′s communicating that he doesn't like change through that behaviour, you know the humming and gauging. Have you seen him put his hand in his mouth, you know the flapping? That happens when we change something.^25^

These descriptions focus on understanding idiosyncratic communication and learning to appreciate the richness of expressions that bodies retain in the absence of verbal language.^21^ Inherent in these are also a focus upon the ‘other’, thus highlighting the relational aspect:

The role of supporters within this dynamic is to respond to this expression of will and preference by acknowledging, interpreting and responding to it.^25^

## PHILOSOPHICAL AND ETHICAL REASONING

Implicit in the aim of understanding and supporting a person towards self‐determination is a view that people with PIMD have moral values and should be treated as such, thus counteracting perspectives that these are people with lower moral worth.^11^ This might be grounded on human rights:

Although that approach towards broadening the idea of self‐determination poses significant risks, it follows from strict obligations to human rights.^23^

One of the included articles, by Kittay,^26^ argues for the personhood of people with PIMD, stating that rationality as the ultimate human trait has failed, and that it is rather our relational coexistence that moral worth is grounded on. The literature argues that recognizing the human condition of dependency should not thereby dismiss moral value, but rather recognize that humans are interdependent and embodied.^21^, ^23^ Relationships marked by such dependency are still embedded with reciprocity:

Inaaya is still a child, still a child, and as much a person as you and me … I look at how beautiful her eyes are, her eyelashes are, how her hands are, how she feels my arm or leans back on me – that's her way of giving, her way of reciprocating.^27^

Following from this is a perspective that challenges knowledge as individualistic, instead viewing meaning as co‐constructed^22^, ^23^ through trusting relationships^21^ and a broad interpretation of communication.^25^

… understanding about individuals' modes of communication and preferences concerning interaction can only be built over time, through sustained presence and participation in their lives.^21^

Boahen^20^ points to the ethical value of highlighting the strengths and capabilities people with PIMD possess, a perspective sometimes missing when classifying such individuals in deficient terms. The two philosophical articles^26^, ^27^ suggest that essential assumptions about human dependency of people with PIMD are relevant to all people.

## LEARNING FROM THE PEOPLE WITH PIMD

Through the examples of real‐life cases merging with certain philosophical and ethical views, knowledge relevant to self‐determination is extracted, both about how to listen and learn from individuals' experiences and about what these add to our understanding of self‐determination.

The first perspective, how to listen and learn, involves recognizing the condition of mediating support that hallmarks the lives of people with PIMD,^23^ inviting both researchers and practitioners to be modest about what they can understand and urging them to reflect upon validity issues when aiming to grasp the embodied life of another. The necessary interpretation is imbued with uncertainty and ambiguity, and despite being close to another's life through ethnographic endeavours, the potential for misinterpretations is extensive. With this in mind, researchers may still propose suggested understandings, but in ways sensitive to what unfolds from that individual's lived experience. Giving room for ethnographic description rather than definitive conclusions may be fruitful. It may also be rewarding to accept the possible questions that might not be disclosed, allowing space to ponder the descriptions of the life of the person with PIMD. Another implication of mediation is viewing meaning as something that can emerge as co‐constructed. Although one could argue for the importance of letting a person demonstrate opinions in individual, independent ways, for example through the use of different technical devices, the ethnographies in this review demonstrate how meaning occurs through intimate interactions, marked by negotiations and partnership. In these encounters, the person with PIMD calls us – not just researchers but also professionals providing services – to act in sensitive, ethical ways, paying attention to the person's embodied, unique communication.

Regarding the second perspective, the issue of self‐determination, the main message is that all people have the potential for self‐determination. This point is demonstrated by advancing the concept. Through the condition of dependency, the descriptions of lived life demonstrate moments of self‐determination, namely living a life according to one's own preferences, through sensitive relations with others. These real‐life descriptions call for approaching self‐determination as more than mere choice‐making, viewing it instead as a continuous process of being understood.^23^, ^25^, ^27^ This process involves a great responsibility on the part of the professional in enhancing self‐determination,^24^ thus emphasizing a delicate dynamic: all situations that might lead to the person experiencing congruence might also lead to ignoring or overlooking that potential.^23^, ^24^ The encounters with people with PIMD suggest certain essentials of this relationship, wherein fostering self‐determination demands relational and communicative sensitivity. The literature argues for the importance of supporter responsiveness and relational closeness,^25^ as well as for the importance of recognizing the embedded imbalance in the relationship.^21^ When it comes to communication, the literature demonstrates how an embodied sensitivity is necessary to fully appreciate a broadened view of how meaning can be displayed.^21^, ^22^, ^23^, ^24^, ^25^

## CONCLUSION

Different perspectives enhance our understanding of lives that, for many, are hidden and marginalized. Ethnographic descriptions further inform philosophical and ethical understanding, offering insights, both for future research and for clinicians working with people with PIMD. Specifically, it is important to develop sensitivity to listen to and learn from embodied ways of being. Encounters with people with PIMD invite us to understand both human rights and philosophical issues in new ways, calling attention to what is missing from the person's account. Such research demands a sensitivity to the singularity of each person. Furthermore, the perspective of people with PIMD demonstrates the human condition of dependency—an insight relevant for all humans.

## Supporting information


**Figure S1**: Flow chart of literature selection process.

## Data Availability

Data sharing not applicable ‐ no new data generated
