# Supplementary material for: Self‐determination of people with profound intellectual and multiple disabilities
Source: Dev Med Child Neurol. 2022 Jul 22;65(1):16–23. doi: 10.1111/dmcn.15363 (PMC10084087; doi:10.1111/dmcn.15363)
Supplement: Supplementary file 1 — Figure S1: Flow chart of literature selection process. [file DMCN-65-16-s001.pdf]

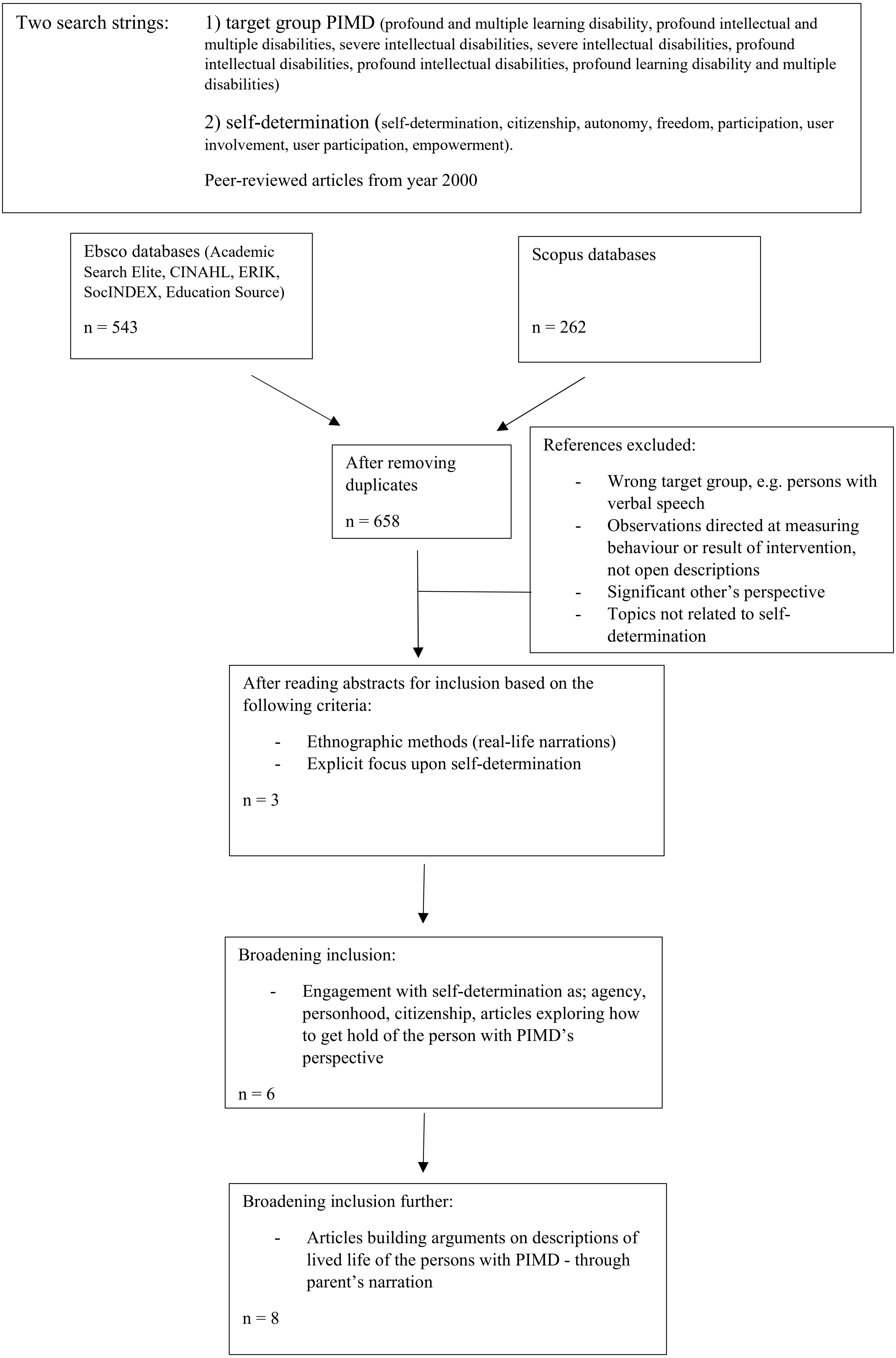

Figure S1: Flow chart of literature selection process
